# Performance of a fully‐automated system on a WHO malaria microscopy evaluation slide set

**DOI:** 10.1186/s12936-021-03631-3

**Published:** 2021-02-25

**Authors:** Matthew P. Horning, Charles B. Delahunt, Christine M. Bachman, Jennifer Luchavez, Christian Luna, Liming Hu, Mayoore S. Jaiswal, Clay M. Thompson, Sourabh Kulhare, Samantha Janko, Benjamin K. Wilson, Travis Ostbye, Martha Mehanian, Roman Gebrehiwot, Grace Yun, David Bell, Stephane Proux, Jane Y. Carter, Wellington Oyibo, Dionicia Gamboa, Mehul Dhorda, Ranitha Vongpromek, Peter L. Chiodini, Bernhards Ogutu, Earl G. Long, Kyaw Tun, Thomas R. Burkot, Ken Lilley, Courosh Mehanian

**Affiliations:** 1 Global Health Labs (formerly at Intellectual Ventures Laboratory/Global Good), 14360 SE Eastgate Way, Bellevue, WA 98007 USA; 2 Applied Math Department, University of Washington, Seattle, WA 98195 USA; 3 formerly Intellectual Ventures Laboratory, 3150 139th AVE SE, Bellevue, WA 98005 USA; 4 Research Institute for Tropical Medicine, Muntinlupa, Philippines; 5 Creative Creek LLC, Camano Island, WA USA; 6 Arizona State University, Tempe, AZ USA; 7 Independent Consultant, Issaquah, WA USA; 8 Shoklo Malaria Research Unit, Mahidol Oxford Tropical Medicine Research Unit, Faculty of Tropical Medicine, Mahidol University, Mae Sot, Thailand; 9 Amref Health Africa, Nairobi, Kenya; 10 University of Lagos, Lagos, Nigeria; 11 Laboratorios de Investigacion y Desarrollo, Facultad de Ciencias y Filosofia, Universidad Peruana Cayetano Heredia, Lima, Peru; 12 World Wide Antimalarial Resistance Network and Mahidol-Oxford Tropical Medicine Research Unit, Bangkok, Thailand; 13 Infectious Diseases Data Observatory and World Wide Antimalarial Resistance Network, Asia- Pacific Regional Centre, Bangkok, Thailand; 14 Hospital for Tropical Diseases and the London School of Hygiene and Tropical Medicine, London, UK; 15 Kenya Medical Research Institute, Nairobi, Kenya; 16 Centers for Disease Control and Prevention, Atlanta, GA USA; 17 Defence Services Medical Academy, Mingaladon, Myanmar; 18 Australian Institute of Tropical Health and Medicine, James Cook University, Cairns, Australia; 19 Australian Defence Force Malaria and Infectious Disease Institute, Enoggera, Australia

**Keywords:** Malaria, Automated diagnosis, Machine learning, Microscopy, WHO

## Abstract

**Background:**

Manual microscopy remains a widely-used tool for malaria diagnosis and clinical studies, but it has inconsistent quality in the field due to variability in training and field practices. Automated diagnostic systems based on machine learning hold promise to improve quality and reproducibility of field microscopy. The World Health Organization (WHO) has designed a 55-slide set (WHO 55) for their External Competence Assessment of Malaria Microscopists (ECAMM) programme, which can also serve as a valuable benchmark for automated systems. The performance of a fully-automated malaria diagnostic system, EasyScan GO, on a WHO 55 slide set was evaluated.

**Methods:**

The WHO 55 slide set is designed to evaluate microscopist competence in three areas of malaria diagnosis using Giemsa-stained blood films, focused on crucial field needs: malaria parasite detection, malaria parasite species identification (ID), and malaria parasite quantitation. The EasyScan GO is a fully-automated system that combines scanning of Giemsa-stained blood films with assessment algorithms to deliver malaria diagnoses. This system was tested on a WHO 55 slide set.

**Results:**

The EasyScan GO achieved 94.3 % detection accuracy, 82.9 % species ID accuracy, and 50 % quantitation accuracy, corresponding to WHO microscopy competence Levels 1, 2, and 1, respectively. This is, to our knowledge, the best performance of a fully-automated system on a WHO 55 set.

**Conclusions:**

EasyScan GO’s expert ratings in detection and quantitation on the WHO 55 slide set point towards its potential value in drug efficacy use-cases, as well as in some case management situations with less stringent species ID needs. Improved runtime may enable use in general case management settings.

## Background

Microscopic examination of Giemsa-stained blood films continues to be widely used for parasitological confirmation of malaria diagnosis, representing roughly half of all malaria diagnostic tests performed worldwide in 2018 [1]. Assessments of the therapeutic efficacy of anti-malarial drugs depend on microscopy for the detection and identification of malaria parasites and for estimation of parasite density. However, difficulties in maintaining consistent access to training, quality assurance, and material resources can lead to high variability in the quality of microscopy, hindering programmatic and research operations in the malaria endemic regions where they are most needed [2, 3].

Malaria microscopy is thus a high-value target for automated image-processing and machine learning (ML) systems because such systems can potentially be widely deployed, mitigating the expert-training bottleneck, and because their results are reproducible. Since a thorough review in 2018 [4], there have been several additional reports proposing or evaluating systems for automated interpretation of malaria blood films [5–14].

A lack of benchmark datasets hampers the evaluation and comparison of various automated systems [4, 5]. One strong candidate for such a benchmark is the slide set used for the World Health Organization (WHO) External Competence Assessment of Malaria Microscopists (ECAMM) programme. These slide sets consist of 55–56 carefully specified Giemsa-stained blood films, used to evaluate microscopists in detection, species ID, and quantitation of malaria parasites as part of the WHO Quality Assurance programmes [15, 16]. Assessment based on an ECAMM slide set can offer valuable insight into a system’s effectiveness, because it focuses on the key requirements for viability in field use-cases. Crucially, a system that hopes to deploy through existing field infrastructure, clinicians, and protocols needs to operate successfully on standard Giemsa-stained blood films as currently prepared and processed in the field by microscopists.

This paper reports performance of a fully-automated end-to-end malaria diagnostic system, the Motic EasyScan GO [17], on an ECAMM slide set. The system includes (i) an automated bright-field microscopy platform for scanning of Giemsa-stained thick and thin blood films, and (ii) malaria detection algorithms, previously described in [5, 6], that process the image sets to give parasite detection, species ID, and parasite quantitation at the patient level.

## Methods

### ECAMM test set

**Fig. 1:**
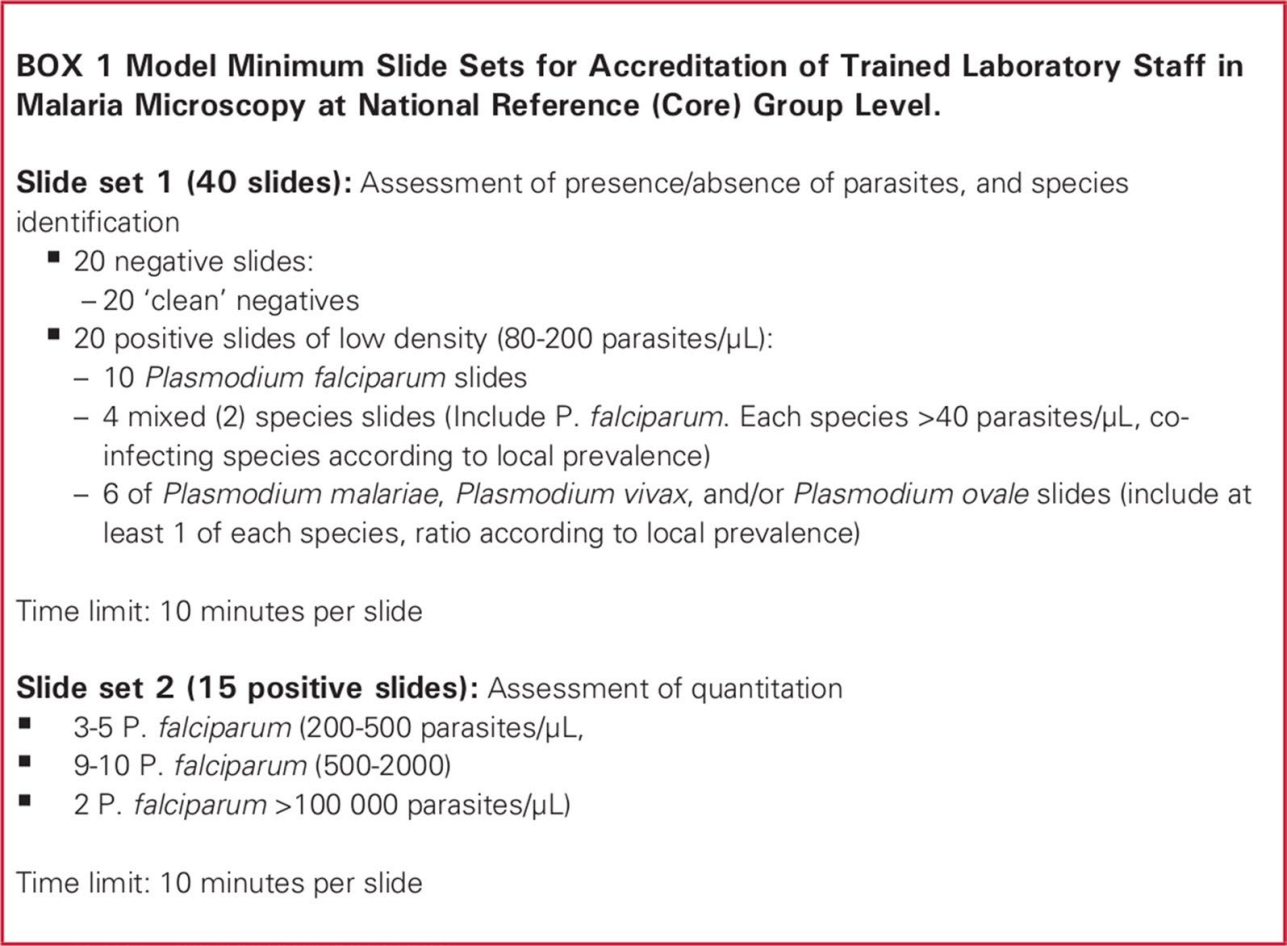
Contents of ECAMM evaluation slide set. From [16]

A set of Giemsa-stained blood films consisting of both malaria positive and negative cases and varying parasite densities were obtained from the WHO WPRO (Regional Office for the Western Pacific) regional malaria slide bank, a collection of reference slides managed by the WHO Collaborating Centre for malaria diagnosis at the Research Institute for Tropical Medicine (RITM) [18]. All slides in the slide bank were previously validated by 12 independent microscopists certified as Level 1 malaria microscopists through the WHO ECAMM. Each microscopist read two representative slides from a set of about 200 slides per case. The parasite species was confirmed by at least 70 % of the readers and by polymerase chain reaction (PCR). Parasite counts per microliter were estimated against 500 white blood cells and calculated with an estimated average white cell count of 8000/*µ*L, and the median of 24 readings was taken as the reference count. Slides with statistically significant inter- or intra-reader count variation are never selected for the ECAMM sets. The 55-slide set used in the experiments for this study was assembled by WHO WPRO to represent the template for ECAMM slide sets described in [16] and shown in Fig. 1 (henceforth the WHO 55). However, it had a few deviations. Such deviations are common in ECAMM workshops (K. Lilley, pers. commun.) due to the logistical difficulties of assembling an ideal set. The deviations present in this set are described below, and their effects on the results are discussed in the Discussion section. For detailed contents of the set used in this study, see Additional File 1.

The ECAMM slide set is designed to evaluate a microscopist’s competence in three key areas important for malaria diagnosis: (1) malaria parasite detection, (2) malaria parasite species ID, and (3) quantitation of malaria parasites. Accuracy in each of these areas is evaluated using a different subset of the ECAMM slide set (see [16] and Fig. 1):

#### Detection

Ideally, the detection component uses 20 negative samples and 20 positive samples with a parasitaemia ranging from 80 to 200 parasites per microlitre (p/*µ*L). Given the samples in the set provided, the *Plasmodium falciparum* samples with a parasitaemia below 200 p/*µ*L (7 slides, rather than 10) and all non-*falciparum* and mixed-species samples (8 slides rather than 10) were used to evaluate detection. The non-*falciparum* slides ranged from 164 to 10,184 p/*µ*L with most samples above 1000 p/*µ*L, well above the ideal range of 80–200 p/*µ*L.

#### Species identification

Ideally, the species ID component uses the same samples, 20 negative and 20 positive, as the detection subset. Negative samples are used along with positive samples for evaluating species ID accuracy during ECAMM (K. Lilley, pers. commun.). The same subset of slides, 20 negative and 15 positive, used for Detection were used for Species ID. The provided set also contained only one mixed-species sample, rather than four as called for by the WHO malaria microscopy quality assurance manual [16].

#### Quantitation

Ideally, the quantitation component uses 15 *P. falciparum* slides with parasitaemias within 200 ≤ p/*µ*L ≤ 2000, plus one or two very high parasitaemia slides. From the provided set, the 20 *P. falciparum* slides with parasitaemia *>* 200 p/*µ*L (rather than 15) were used. Of these, 18 slides had 200 ≤ p/*µ*L ≤ 2000, and two slides had ∼200,000 p/*µ*L. An estimated parasitaemia within 25 % of the reference value was considered correct, per the WHO malaria microscopy quality assurance manual [16]. The parasitaemia range is important, because slides with low parasitaemias have higher Poisson variability and are thus harder to quantitate accurately (as provided in the supplementary information of reference [19]).

A newer, slightly modified version (“V2”) of the ECAMM slide set template, with detailed protocols on its use during evaluation of microscopists, is found in [15]. “V1” [16] was used in this study, because (i) the provided ECAMM set was designed to match “V1”; and (ii) the “V2” protocols describe using a subset of the slides during an orientation phase prior to testing, which does not align with standard machine learning practice (the samples used to test an algorithm should never be used during training).

### Slides used for algorithm development

The malaria detector algorithm was trained and validated using over 500 slides from 11 countries. Sources of training data (with geographic source of the slides in parentheses) included Shoklo Malaria Research Unit (Thailand), Amref Health Africa (Kenya), University of Lagos (Nigeria), Universidad Peruana Cayetano Heredia (Peru), World Wide Antimalarial Resistance Network (Thailand, Indonesia, Cambodia, and DR Congo), Hospital for Tropical Diseases and the London School of Hygiene and Tropical Medicine (UK), Kenya Medical Research Institute (Kenya), Centers for Disease Control and Prevention (USA and other countries), James Cook University (Solomon Islands), and the Defence Services Medical Academy (Myanmar).

No slides from RITM were used for training the machine learning algorithm models. A separate set of 48 slides from RITM, also roughly analogous to an official ECAMM set, was used along with the other slides listed above to tune final diagnostic parameters in the patient-level disposition step, prior to any exposure to the ECAMM test set.

### Imaging method

The slides, permanently mounted with cover glass, were scanned with a prototype version of the Motic EasyScan GO [17]. The microscope had a 40×, NA = 0.75 objective, infinity-corrected optical train, and 10 W LED Köhler illumination. A CMOS camera captured images (2048 × 1536 pixels) at an approximate pixel pitch of 8.3 pixels/*µ*m at the sample plane. Each field-of-view (FoV) was captured as a stack of 5 slices with 0.5 *µ*m vertical spacing, to ensure in-focus thumbnails of all objects-of-interest (e.g. parasites), which can lie at different depths due to the thickness of the blood films and/or slight tilting of the microscope slide.

On the thick film, a 20 × 20 grid of FoVs near the centre was manually selected and scanned. This gave an area scanned of roughly 18 mm^2^, equivalent to ∼580 fields of view on a manual microscope with 100× oil immersion lens and 20 mm field number eyepiece, yielding ∼3 k to 17 k white blood cells (WBCs).

On the thin film, a rectangular region near the “feathered edge”, where a monolayer of red blood cells (RBCs) is most often found, was manually selected and scanned, yielding 150 to 192 FoVs and ∼10 k to 50 k non-overlapped RBCs. The area selected was based only on the presence of a monolayer of RBCs and not on whether parasites were visible in the region.

### Algorithm

The image sets were analysed by both thick film and thin film malaria detector algorithms that detect and evaluate objects of interest. A final synthesis block combined the thick and thin film findings to produce a diagnosis report indicating: (i) whether malaria parasites were detected; (ii) the suspected malaria species; and *(iii)* the total parasite count (also ring-stage and late-stage counts) given as parasites per 8000 WBCs. This quantitation unit allows direct comparison to standard methods that assume a WBC density of 8000/*µ*L, but also allows for correction when the WBC count is known.

To assess performance on the ECAMM set, the results of the algorithm on each slide were compared with the reference readings from RITM, and performance was evaluated according to the rubric in the WHO malaria microscopy quality assurance manual [16] (shown in Table 1).

**Table 1:** Scoring rubric for ECAMM evaluation slide set, from [16]

Accreditation level	Detection	Species identification	Parasite quantitation
Based on lowest grade achieved			(within 25% of reference count)
1	≥ 90 %	≥ 90 %	≥ 50 %
2	80 % –<90 %	80 % -–<90 %	40 % –<50 %
3	70 % -–<80 %	70 % -–<80 %	30 % –<40 %
4	< 70 %	< 70 %	< 30 %

**Fig. 2:**
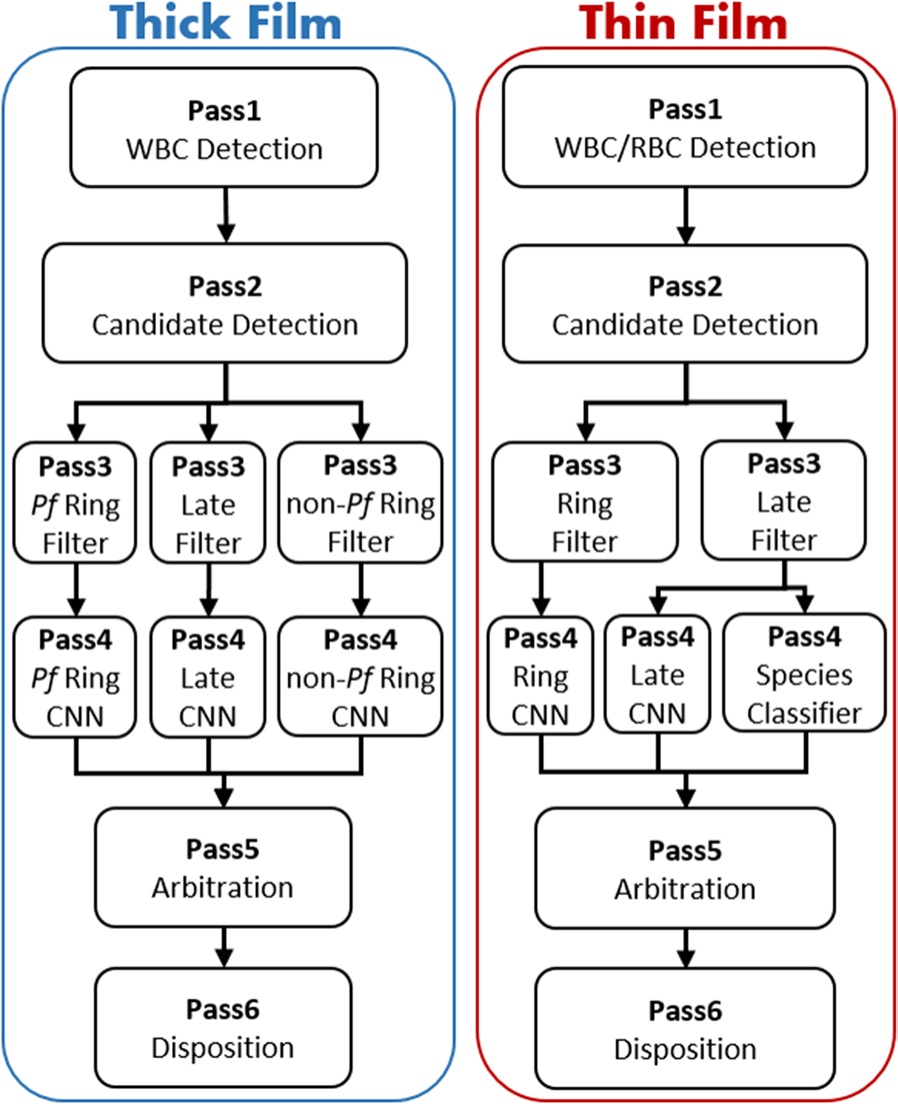
Malaria detector algorithm architecture. The thick film (left) and thin film (right) malaria detector algorithms each consist of six passes. Multiple branches, each with a paired Pass3-Pass4 block, target different parasite morphologies. A synthesis module (not shown) combines the thick and thin film findings to give a patient diagnosis

A schematic representation of the algorithm modules is shown in Fig. 2. Full algorithm details are found in [6] for thick film, and [5] for thin film. This section (i) provides a brief overview of the two algorithms; (ii) notes the updates to the current thick film algorithm from the version in [6]; and (iii) describes the synthesis block that combines thick and thin film findings to give the final slide diagnosis.

#### Algorithm overview

Both algorithms consist of various processing blocks as depicted in Fig. 2. The term “Pass” refers to the different modules of processing (vertically laid out in Fig. 2) from *Pass1* at the top to *Pass6* at the bottom. The term “branch” refers to multiple blocks in *Pass3*, *Pass4* processing (*Pf* Ring, Late, non-*Pf* Ring, horizontally laid out in Fig. 2). The processing performed in *Pass1* through *Pass6* is described below.

##### Pass1

 Analyses every FoV stack for quality and focus. WBCs and RBCs are detected at the best focus level for each FoV. Thin film RBC and WBC detection use traditional computer vision techniques (e.g. thresholding, segmentation, feature extraction, classification), whereas the thick film uses a region-based convolutional neural network (CNN), Faster R-CNN [20], for WBC detection.

##### Pass2 

Finds candidate parasites in all FoVs at all focus levels, via traditional computer vision techniques. The most in-focus thumbnail of each candidate object is selected for further processing.

##### Pass3

 Weeds out obvious non-parasites (distractors) via traditional feature extraction and a gradient-boosted decision tree classifier [21].

##### Pass4

 Applies a CNN classifier to thumbnails of all candidate parasite objects that survived the distractor filter of *Pass3*. In thin film only, a species classifier is applied to late-stage objects in a species branch [5].

##### Pass5

 Arbitrates the appropriate stage type (ring or late) of objects detected by multiple branches, using the highest CNN score.

##### *Pass6*

(Disposition) processes all the detected and classified objects to (i) determine whether the blood film (thick or thin) is overall positive for malaria; (ii) estimate the parasitemia if positive; and (iii) determine the species of malaria if positive (potentially more than one).

##### Branches

Multiple branches of paired *Pass3-Pass4* modules are applied to the detected candidate objects. Each branch is trained and tuned to detect a different life-cycle stage of the malaria parasite. The ring-stage branch targets young ring-form trophozoites (in thick film only, there are separate *P. falciparum* and non-*falciparum* ring-stage branches). The late-stage branch targets late-stage trophozoites, schizonts, and gametocytes, which are common in non-*falciparum* species and much rarer in *P. falciparum*. The species branch (thin film only) extracts a set of manually designed features for species ID, then classifies objects by species and stage via a gradient-boosted decision tree classifier.

#### Thick film algorithm updates

The thin film algorithm used in this study is identical to the one reported in [5]. The thick film algorithm is largely the same as the one reported in [6], but has been further developed:

(i) The algorithm in [6] was trained on images acquired with a prototype scanning microscope, whereas the results reported here are based on images scanned with the EasyScan GO [17].
(ii) The WBC detector is now based on a CNN object detection architecture, Faster R-CNN [20], as opposed to traditional computer vision techniques.
(iii) The distractor filter of *Pass3* has been enhanced by doubling the number of discriminative features.
(iv) The thick-film algorithm has a new third detection branch targeting non-*falciparum* ring-stage trophozoites. This helps distinguish *P. falciparum* from non-*falciparum* infections solely from the thick film, even if a non-*falciparum* sample presents few or no late-stages, by recognizing that beyond the youngest ring-stages, non-*falciparum* and *P. falciparum* trophozoites differ morphologically.
(v) The *Pass6* disposition block has an added function that assesses whether a thick film slide may be poorly prepared, based on the presence of very large numbers of distractor objects. If this condition is detected, the disposition block can adjust the diagnosis thresholds to reduce the chance that a malaria-negative but debris-filled slide will be diagnosed as malaria-positive.

#### Synthesis block

The thick and thin film algorithms are independent and can provide conflicting results for the same sample. The synthesis block combines the two findings as follows. It uses the thick film analysis to determine three results:

(i) Malaria diagnosis (i.e. whether malaria parasites are present). This matches standard microscopy practice [22].
(ii) Quantitation of parasites, at all parasitaemias. This differs from microscopy practice, which recommends switching to the thin film when the density on thick films is very high (roughly 80,000 p/*µ*L) [23] due to the difficulty of manually tracking large numbers of parasites in a single FoV. Because an automated system can readily track these higher counts, it can leverage the much larger blood volume on thick films to avoid Poisson variability in parasite counts (as provided in the supplementary information of reference [19]).
(iii) Determination of whether the species is *P. falciparum*, non-*falciparum*, or a mixed infection. This depends on the ratios between the numbers of late-stage, *P. falciparum* ring-stage, and non-*falciparum* ring-stage parasites as well as the total ring-stage count. Mixed infections are identified by very high ring-stage counts (ascribed to *P. falciparum*, since non-*falciparum* species typically have lower parasitaemias) combined with sufficiently high late-stage counts (since *P. falciparum* typically presents very few late-stages).

The synthesis block only uses the thin film results if the thick film algorithm reports a non-*falciparum* or mixed species infection. Thin film results then determine the non-*falciparum* species, as in standard microscopy [22]. If the thick film result is non-*falciparum* or mixed species and the thin film result is negative, the species is predicted as *P. vivax* due to *P. vivax* being the most prevalent non-*falciparum* species of malaria globally [1].

The final report gives three parasitaemias: ring-stage (immature trophozoite), late-stage (mature trophozoite, schizont, and gametocyte), and total. It does not differentiate between the various late-stages. To evaluate quantitation accuracy on ECAMM *P. falciparum* slides, the ring-stage count was used rather than the total parasite count. This is because RITM’s reference readings counted only asexual stages (i.e. non-gametocytes) consistent with malaria microscopy standards [22], and because non-gametocyte late-stages are uncommon in *P. falciparum* blood films.

The final report lists malaria species, WBC count, RBC count (thin film only), and poor slide quality warnings if indicated. It also includes a mosaic of high-scoring objects’ thumbnails for possible examination by the clinician.

## Results

The EasyScan GO malaria detector correctly detected whether or not malaria was present in 33 of 35 slides (20 negative slides and 15 positive slides), for 94.3 % accuracy. It achieved 82.9 % accuracy for species ID, correctly labeling 29 of 35 slides. It correctly quantitated 10 of 20 slides, for 50 % accuracy.

These results correspond to a Level 1, Level 2, and Level 1 microscopist for diagnosis, species ID, and quantitation, respectively, according to the rubric in [16] (shown in Table 1). A microscopist’s final rating equals their lowest-scoring component, so the EasyScan GO would officially receive Level 2 microscopist status. These results are summarized in Table 2. The outputs from the malaria detector for each sample are given in Additional File 1. The results for each section are described in detail below.

**Table 2:** Performance of the EasyScan GO on the various components of the External Competence Assessment slide set

Component of assessment	Number of slides in subset	Number correct	Percentage correct	WHO level on this component
Detection	35	33	94.3	1
Species ID	35	29	82.9	2
Quantitation	20	10	50	1

### Parasite detection

The malaria detector correctly identified whether or not malaria was present in 33 of 35 slides in the detection subset. All negative slides were classified as negative (100 % specificity), and 13 of 15 malaria positive slides were classified as positive (86.7 % sensitivity). This yielded an accuracy of 94.3 % (93.3 % balanced accuracy). The two false negative samples were *P. falciparum* samples with reference parasitaemias of 112 p/*µ*L and 175 p/*µ*L. Also, all 20 *P. falciparum* quantitation slides were correctly classified as positive.

### Species ID

The species was correctly labeled for 29 of the 35 slides (82.9 %) used for evaluating species identification accuracy. This included correctly labelling all 20 (100 %) negative samples, and 9 of 15 (60 %) positive samples, for a balanced accuracy of 80 %. Two errors were due to misclassifying 2 of the 7 *P. falciparum* samples as negative. Also misclassified were 1 out of 3 *Plasmodium malariae* samples, both *Plasmodium ovale* samples, and the single mixed species (*P. falciparum* plus *P. ovale*) sample. A confusion matrix is shown in Table 3, which also gives (in parentheses) the performance on the 20 *P. falciparum* quantitation slides. Of the 20 quantitation slides, 19 were correctly classified as *P. falciparum* and one was incorrectly classified as *Plasmodium vivax*.

Performance on the simpler task of identifying *P. falciparum* versus non-*falciparum* was higher. When combining all 27 *P. falciparum*-only samples in the 55-slide set, 24 were classified as *P. falciparum*, 1 was classified as *P. vivax*, and 2 were classified as negative (88.9 % accuracy). Of the 7 non-*falciparum* samples, 1 was classified as *P. falciparum* and the remaining 6 were classified as non-*falciparum* species (85.7 % accuracy). The mixed sample was classified as *P. vivax*.

**Table 3:** Confusion matrix comparing the reported EasyScan GO species to the reference species. Values in parentheses are the results for slides not used for evaluating species identification accuracy

EasyScan GO species	Reference species					
	*P. falc.*	*P. vivax*	*P. malariae*	*P. ovale*	Negative	mixed *Pf + Po*
*P. falciparum*	5 (19)	–	–	1	–	–
*P. vivax*	− (1)	2	1	–	–	1
*P. malariae*	–	-	2	1	-	–
*P. ovale*	–	-	–	0	–	–
Negative	2	-	–	-	20	–

### Quantitation

**Fig. 3:**
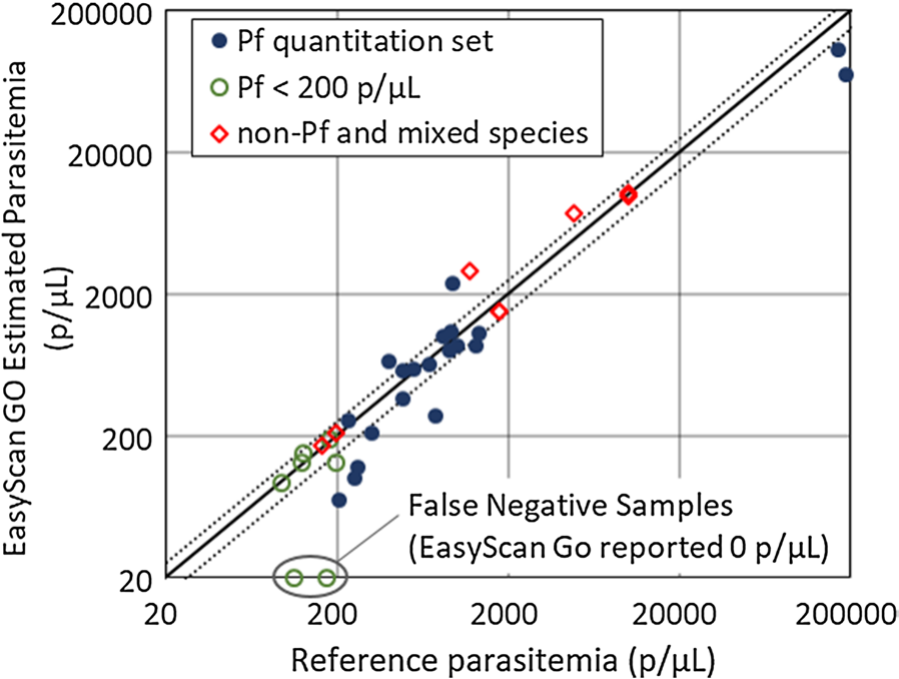
Quantitation accuracy. EasyScan Go quantitation estimate vs. the reference quantitation. For *P. falciparum* samples, the ring-stage count is plotted, whereas for non-*P. falciparum* samples the total count is plotted. The dotted lines show ± 25 % error

Of the 20 *P. falciparum* slides used to evaluate quantitation, the EasyScan GO reported parasitaemias within 25 % of the reference (manually counted) parasitaemias on 10 samples (50 %). Figure 3 plots the EasyScan GO parasitaemia vs. the reference parasitaemia for this set, as well as for the additional positive samples used for detection and species ID. Estimated parasitaemias were within 25 % of reference parasitaemias for 4 of 7 (57.1 %) sub-200 p/*µ*L *P. falciparum* samples, and 6 of 8 (75 %) non-*falciparum* or mixed species samples.

### Timing and invalid results

#### Timing 

The mean time to scan and analyse each slide in the configuration used for this assessment totalled 54.4 minutes: 12.8 and 5.8 minutes for scanning the thick and thin films respectively, and 28.7 and 7.0 minutes for analysing the thick and thin films, respectively. This is well above the 10 minutes per slide allowed in the WHO malaria microscopy quality assurance manual [16].

#### Invalid results

The system is designed to reject a film scan if there are not enough in-focus FoVs, too few WBCs (thick film), or too few RBCs (thin film). On all slides both thick and thin films were successfully scanned, with two exceptions. One thick film was rejected because every FoV was out-of-focus, but was re-scanned successfully. One thin film was rejected because all FoVs were reported as out-of-focus. Upon review, the entire thin film had a very dark background whether viewed with the EasyScan GO or with a standard manual microscope. An example FoV is shown in Additional File 2. Re-scanning was unsuccessful, because the malaria detector could not evaluate focus on the dark background. Since this sample’s thick film was reported as *P. falciparum*, the lack of a thin film result did not affect its final disposition or the above analysis of accuracy.

## Discussion

### WHO 55 as benchmark

The WHO 55 is a template for slide sets used to evaluate malaria microscopist competence (ECAMM) as part of WHO’s quality assurance program. Besides having a consistent composition, it assumes use of existing field infrastructure (i.e. field-prepared Giemsa-stained blood films), and emphasizes (in both content and scoring rubric) the key performance requirements of the most important use-cases. Thus, it may serve as a valuable benchmark dataset to assess and compare automated malaria detection systems. Such benchmarks are currently lacking.

In this paper, the performance of a fully-automated malaria diagnosis system, the EasyScan GO, on such a WHO 55 set is presented. The system scanned Giemsa-stained thick and thin films with an EasyScan GO automated microscope, then applied image-processing and ML methods to generate a slide-level diagnosis.

There are some limitations to using the WHO 55 template as a benchmark for testing automated systems. Each ECAMM slide set is a unique instance of the template, and performance on the set is only a single snapshot of system performance. The slide set is also fairly small, resulting in low statistical power. Ideally, a system should be tested on several ECAMM-style slide sets and should achieve Level 1 on most or all of them as confirmation of its performance. The ECAMM slide sets are also generally of high quality, and do not represent the high variability in sample preparation possible in the field. Internal evaluations (e.g. [5, 6]) and field trials (e.g. [7, 8], as well as ongoing studies) are also vital measures of system performance.

### Diagnosis

The system achieved Level 1 performance on diagnosis of low-parasitemia slides, with 94.3 % accuracy, 86.7 % sensitivity, and 100 % specificity. In the ECAMM slide set used, the non-*falciparum* slides had higher-than ideal parasitaemias. However, it has been observed (in prior experiments and field trials) that late-stage parasites of non-*falciparum* samples are much easier to detect and distinguish from distractor objects than are *P. falciparum* ring-stages, and the algorithm has consistently posted lower limits of detection for late-stages than for ring-stages, making non-*falciparum* samples easier to diagnose at low parasitaemias than *P. falciparum* samples. It is, therefore, likely that the higher parasitaemias on non-*falciparum* slides did not impact the system’s sensitivity results.

### Quantitation

The system also achieved Level 1 performance on quantitation of the 20 relevant *P. falciparum* samples, indicating its potential for use in new drug evaluation and drug resistance studies where accurate estimation of parasite densities is critical.

On the two high parasitaemia samples (∼200 k p/*µ*L), the system’s estimates (done on thick film) were low relative to the reference counts (done on thin films [22]). Given a perfect detector, quantitations from thick films are intrinsically more consistent because the higher volume of blood reduces unavoidable Poisson variability. This is a case where machines have a distinct advantage over even expert humans because of their ability to screen many FoVs without fatigue and track large numbers of objects without error.

### Species ID

The system achieved Level 2 performance on species ID. However, this score was likely inflated due to the differences in the ECAMM test set used compared to the ideal ECAMM template: there were fewer positive samples (15 vs. 20), the parasitaemia of samples was higher than 200 p/*µ*L for 6 (non-*falciparum* and mixed species) samples, and only one sample was mixed species instead of four. Had the test set matched the template, the system likely would have achieved Level 3 due to poor performance on mixed species samples and perhaps reduced performance on lower parasitaemia non-*falciparum* samples. The system’s low species ID rating points to two weaknesses:

(i) Machine learning algorithms are often data-hungry [24], requiring far more training samples than humans do. The training sets were rich in *P. falciparum* and *P. vivax*, but contained few *P. ovale* and *P. malariae* samples since these are less common in malaria-infected humans. *Plasmodium vivax* training samples sufficed to ensure strong *P. falciparum vs.* non-*falciparum* classification on thick films, which enabled the system to have high accuracy at the level of *P. falciparum vs.* non-*falciparum*. But the *vivax-ovale-malariae* classifier suffered from lack of training data.
(ii) The system diagnoses mixed infections by leveraging priors on likely parasitaemias and ring-stage to late-stage ratios of the various species (non-*falciparum* samples tend to have lower maximum parasitaemias and *P. falciparum* late-stage counts tend to be low [25]). It has no way to distinguish mixed infections on low parasitaemia samples, because young ring-stage parasites look similar for all species. So in the absence of a distinctive *P. falciparum* gametocyte, any low-parasitaemia sample containing late-stages is classified as non-*falciparum*. Indeed, the ability of human microscopists to correctly classify such samples is a testament to their skill.

Fortunately, fine-grained species ID is a secondary need in some use-cases. One example is drug resistance studies which involve only quantitation of *P. falciparum* to generate parasite clearance curves. Additionally, geographic priors often indicate the likely type of non-*falciparum* infection. Examples include: *(a)* In South America the two dominant species are *P. vivax* and *P. falciparum* [26], and Peruvian protocols allow use of thick films for species ID [27]; and *(b)* in West Africa, innate resistance to infection with *P. vivax* makes it rare compared to *P. ovale* [28]. The ECAMM template itself specifies incorporating “local prevalence” [15, 16]. However, given travel and other considerations, these geographic priors are not certainties.

Species ID is important for some treatment decisions [29]. Examples include: *(a) P. falciparum* infection has a high fatality rate, so high *P. falciparum* sensitivity is important; and *(b) P. vivax* and *P. ovale* develop hypnozoites, which require distinct treatment, so accurate *falciparum-malariae* vs. *vivax-ovale* species ID is desirable.

### Thick‐film only evaluation

The system mostly depends on thick film results, for various reasons:

(i) In many situations (e.g. drug resistance sentinel sites, and sites with only one non-*falciparum* species such as Peru), this is sufficient.
(ii) In the field, thin film quality can be highly variable (E. Long, pers. commun.), and thick film-only workflows can be preferable for various reasons (J. Carter, pers. commun.).
(iii) Machine learning algorithms are often data hungry, and owing to the greater density of blood on thick films the training sets contained more parasite images from thick films than from thin films.

However, successfully distinguishing between non-*falciparum* species using only thick films has not yet been achieved and the algorithm still depends on thin films for this task.

### ECAMM versions

The “V2” ECAMM slide set [15] has some minor differences from “V1”. These changes might have affected the system’s performance, in either direction. Also, “V2” protocols specify that 30 % of the slides be used during a preparation or orientation phase, i.e. the slide set is not treated as a true holdout set (a set of samples which is not seen prior to testing) since the microscopists receive some training on the slide set prior to assessment. Such prior exposure, if incorporated into training of an algorithm, would complicate the training flow and would also strongly affect machine results (by the nature of machine learning). In this regard, the “V2” protocols are not as well suited to evaluation of computer-automated systems.

### Timing

Whether the current system runtime is acceptable depends on particular field constraints. In some field clinics, fast throughput is essential, and the system would be inappropriate for such scenarios. In other settings, the ability to analyse batches of samples overnight would mitigate long runtime (e.g. re-checking of slides for quality control).

Since these experiments were run, several changes have been made to the EasyScan GO software to shorten runtime, including:

(i) Scan a lower volume of blood, using thresholds on FoVs, WBCs (thick film) and RBCs (thin film), rather than scanning a fixed number of FoVs. This enables the blood volume scanned to more closely match manual microscopy protocols (100 FoVs with a 100× objective rather than the ∼580 scanned here, and at least 500 WBCs [22], rather than the average of 8000 WBCs scanned here). Analysis time scales roughly linearly with the number of FoVs.

(ii) Analyze thick film first and proceed to thin film analysis only when indicated, i.e. only in case of a positive, non-*falciparum* result.

(iii) Perform scanning and analysis in parallel instead of in series, e.g. a second sample can be scanned while the first sample is being analysed.

Further experiments are needed to characterize the resulting changes in runtime, and to ensure there is no loss in accuracy due to the decreased scan area. Preliminary experiments indicate typical total time of 18–25 minutes per slide with these changes. To simulate the effect on accuracy of reducing FoVs, the samples were re-analysed using 70 FoVs (the equivalent of 100 FoVs using a standard microscope with a 100× objective), selected from the original scanned FoVs, for each thick film. This did not change the detection or species ID results for any sample, however the quantitation results varied by 0.7x to 2.3x compared to the original counts. A detailed discussion is beyond the scope of this report, but these results are included as columns in Additional file 1.

Finally, analysis time is dependent on computing power. This study was performed on a computer with specifications matching that provided as part of the EasyScan Go system – a relatively high-end laptop (as of 2019) with an Intel i7-9750H CPU, an NVIDIA GeForce GTX 1650 GPU, and 16 GB RAM.

### Machine vs. human trade-offs

In malaria microscopy, as in other domains, machines have pros and cons relative to human workers:

(i) In general, machines can run long hours without getting tired, but conversely they are at risk of mechanical failures.
(ii) Once developed, machines can be deployed at scale, without personnel training bottlenecks (although for this application there remains a need for sample collection and preparation by trained personnel).
(iii) Machine learning algorithms are notoriously brittle in the face of test samples that differ markedly from their training data. In the malaria task, algorithm performance can degrade in deployments to new locations if the clinics’ slide preparations have unfamiliar characteristics [7]. In such cases, humans adapt much more readily to the novel characteristics. For example, the thin film described in this report that was rejected by the system was, while highly abnormal, still interpretable by a human.

To develop ML algorithms for malaria is to enjoy ample opportunities to recognize and admire the skill, versatility, rapid learning, and adaptability of human workers. At the same time, EasyScan GO’s strong performance on this benchmark slide set is evidence that fully-automated systems are poised to play a meaningful role in malaria field microscopy.

## Conclusions

Automated (machine learning-based) systems are a promising way to improve the quality and consistency of malaria microscopy. The WHO 55 slide set, designed to evaluate microscopists’ competence in crucial field use-cases, can serve as a benchmark for evaluating such systems. The fully-automated EasyScan GO, a slide scanning microscope coupled with malaria detection algorithms, was evaluated on a WHO 55 slide set. It achieved Level 1 competence in Diagnosis and Quantitation, and Level 2 in Species ID, the best performance on this benchmark test of any fully-automated system to our knowledge. While runtime and species ID both require improvement for use in general case management settings, its strong results on this slide set indicate its potential value for research use-cases such as drug efficacy monitoring, and possibly for case management use-cases with less stringent species ID requirements.

## Supplementary Information

**Additional file 1:**A full list of samples, reference results, and results of the malaria detector algorithms.

**Additional file 2:**An example Field of View from the unreadable thin film, compared to an acceptable FoV.

## Data Availability

The data supporting the conclusions of this article (results from the EasyScan Go) are included within the article and its additional file(s). Requests for access to ECAMM slide sets should be made to the WHO Collaborating Center for Malaria Diagnosis at RITM [18].

## Abbreviations

CNN: Convolutional neural network
ECAMM: External competence assessment of malaria microscopists
FoV: Field-of-view
ID: Identification
ML: Machine learning
PCR: Polymerase chain reaction
RBC: Red blood cell
RITM: Research Institute for Tropical Medicine
WBC: White blood cell
WHO: World Health Organization
WPRO: Regional Office for the Western Pacific
