# Supplementary material for: Performance of a fully‐automated system on a WHO malaria microscopy evaluation slide set
Source: Malar J. 2021 Feb 25;20:110. doi: 10.1186/s12936-021-03631-3 (PMC7905596; doi:10.1186/s12936-021-03631-3)
Supplement: Supplementary file 2 — Additional file 2:An example Field of View from the unreadable thin film, compared to an acceptable FoV. [file 12936_2021_3631_MOESM2_ESM.pdf]

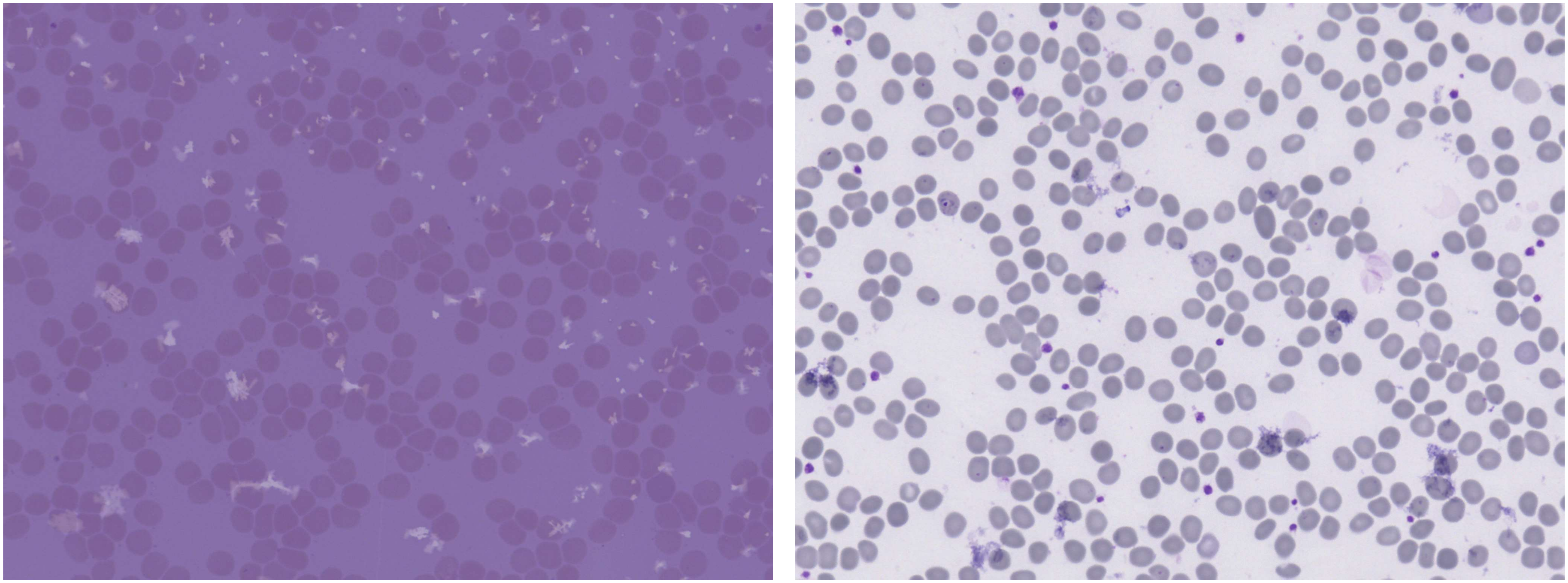

Additional File 2: Example field of view from the thin film rejected by the EasyScan Go malaria detector (left), and an example of a typical thin film field of view (right).
